# Development and evaluation of the MAINTAIN instrument, selecting patients suitable for secondary or tertiary preventive manual care: the Nordic maintenance care program

**DOI:** 10.1186/s12998-022-00424-6

**Published:** 2022-03-17

**Authors:** Andreas Eklund, Per J. Palmgren, Ulf Jakobsson, Iben Axén

**Affiliations:** 1 Unit of Intervention and Implementation Research for Worker Health, The Institute of Environmental Medicine (IMM), Karolinska Institutet, Stockholm, Sweden; 2 Department of Learning, Informatics, Management and Ethics (LIME), Karolinska Institutet, Stockholm, Sweden; 3 Department of Clinical Sciences Malmö, Centre for Primary Health Care Research, Lund University, Lund, Sweden

**Keywords:** MAINTAIN, Clinical instrument, Low back pain, Stratified care, Chiropractic, Prevention, Maintenance care, Manual treatment, Effect, Secondary prevention, Tertiary prevention

## Abstract

**Background:**

Chiropractic maintenance care (MC) has been found to be effective for patients classified as dysfunctional by the West Haven-Yale Multidimensional Pain Inventory (MPI). Although displaying good psychometric properties, the instrument was not designed to be used in clinical practice to screen patients for stratified care pathways. The aim was to develop a brief clinical instrument with the intent of identifying dysfunctional patients with acceptable diagnostic accuracy.

**Methods:**

Data from 249 patients with a complete MPI dataset from a randomized clinical trial that investigated the effect and cost-effectiveness of MC with a 12-month follow-up was used in this cross-sectional analysis. A brief screening instrument was developed to identify dysfunctional patients, with a summary measure. Different cut-offs were considered with regards to diagnostic accuracy using the original instrument’s classification of dysfunctional patients as a reference. Very good diagnostic accuracy was defined as an area under the curve (AUC) metric between 0.8 and 0.9. The instrument was then externally validated in 3 other existing datasets to assess model transportability across populations and medical settings.

**Results:**

Using an explorative approach, the MAINTAIN instrument with 10 questions (0–6 Likert responses) capturing 5 dimensions (pain severity, interference, life control, affective distress, and support) was developed, generating an algorithm-based score ranging from − 12 to 48. Reporting a MAINTAIN score of 18 or higher, 146 out of the 249 patients were classified as dysfunctional with 95.8% sensitivity and 64.3% specificity. At a score of 22 or higher, 109/249 were classified as dysfunctional with 81.1% sensitivity and 79.2% specificity. AUC was estimated to 0.87 (95% CI 0.83, 0.92) and Youden’s index was highest (0.70) at a score of 20. The diagnostic accuracy was similar and high across populations with minor differences in optimal thresholds for identifying dysfunctional individuals.

**Conclusion:**

The MAINTAIN instrument has very good diagnostic accuracy with regards to identifying dysfunctional patients and may be used as a decision aid in clinical practice. By using 2 thresholds, patients can be categorized into “low probability (− 12 to 17)”, “moderate probability (18 to 21)”, and “high probability (22 to 48)” of having a good outcome from maintenance care for low back pain.

**Trial registration:**

Clinical trials.gov; NCT01539863; registered February 28, 2012; https://clinicaltrials.gov/ct2/show/NCT01539863.

**Supplementary Information:**

The online version contains supplementary material available at 10.1186/s12998-022-00424-6.

## Background

Non-specific low back pain (LBP) is a highly prevalent condition affecting a large part of the adult population with major consequences worldwide. It is considered one of the biggest economic burdens in western societies [1]. According to the Global Burden of Disease Study, LBP results in more years lived with disability than any other disease in the world [2]. LBP episodes are often short lived but recurrent throughout life and the lifetime prevalence worldwide has been estimated to be 80–85% [2]. Therefore, it is recommended that research should aim at secondary prevention (preventing recurrences in people recovered from a previous episode) and tertiary prevention (preventing progression and consequences of LBP) [3].

Chiropractic maintenance care (MC) is described as a long-term management strategy for musculoskeletal disorders, introduced when optimum treatment benefit has been reached after an initial care plan. The aim of MC is to prevent future episodes, progression, and consequences of LBP by treating the patient at regular intervals, regardless of symptoms [4–18]. In an ambitious effort, researchers across the Nordic countries have systematically explored and investigated indications, content, and frequency of MC in a series of research projects [18]. Commonly, MC patients are selected based on their previous history of pain and the effectiveness of the initial care plan [10]. Selected MC patients are commonly scheduled with 1–3 months intervals and are treated with manual therapy, along with individual exercises and lifestyle advice [18].

Based on this knowledge, a pragmatic randomized clinical trial (RCT) was designed to investigate the effectiveness of MC in patients with recurrent and persistent LBP [19]. The trial exhibited that the MC-group had fewer days with bothersome LBP over a year compared to the control group [20]. Although more effective, the number of visits was higher in the MC-group with more treatments during the 52-week study period [20]. There was a large variability in the data, suggesting that there may be subgroups of patients who experienced fewer days of pain and fewer visits than others.

Psychological [21, 22], behavioural [23] and social characteristics [24] of LBP patients are important in the transition from acute to recurrent and persistent pain states [25–31]. In line with the bio-psycho-social model, the leading theoretical framework underpinning the management of LBP [24, 32–34] clinicians would be expected to consider cognitive processes, psychological and behavioural dimensions of the pain experienced when managing patients with pain. Based on the cognitive-behavioural conceptualization of pain, The West Haven-Yale Multidimensional Pain Inventory (MPI) was developed to capture the perceptions and consequences of living with chronic pain [35]. The original instrument has been used to identify three clusters/subgroups of patients [36] and has been shown to be reliable, valid, and useful in outcome-based research [37, 38]. The three different subgroups are defined as adaptive copers (AC), interpersonally distressed (ID), and dysfunctional (DYS). Individuals in the AC group are characterized by low pain severity, low interference with everyday life due to pain, low life distress, a high activity level, and a high perception of life control and have the best prognosis with the lowest risk for long-term sick leave [39]. Individuals in the ID group are characterized by dysfunctional behaviours such as low levels of social support, low levels of solicitous and distracting responses from significant others, and high scores on punishing responses compared to the DYS and AC patients [39]. Individuals in the DYS subgroup are characterized by having high pain severity, marked interference with everyday life due to pain, high affective distress, low perception of life control, and low activity levels and have the worst prognosis along with the highest risk of long-term sickness absence [39].

In a secondary analysis of the data from the RCT [40, 41], it was found that patients with a less favourable psychological profile (DYS subgroup) reported better outcomes from the MC approach. Surprisingly, the effect of MC within the DYS subgroup was achieved at an equal number of visits compared to the control group. On the other hand, patients within the AC group who received MC reported more days with pain while also receiving a higher number of visits compared to the control group. These results may change the way MC is delivered in clinical practice as we can now identify subgroups of patients where MC is most effective. At the same time, it becomes clear that for a specific group of patients, the AC group, MC should not be recommended.

Efforts have been made to tailor treatments to specific subgroups of patients with recurrent or persistent LBP [28, 37, 42–48]. However, none of the available instruments have been able to improve outcomes among chiropractic patients by identifying candidates suitable for a stratified care pathway [49, 50]. Previous research has investigated the predictive ability of the Keele STarT Back Tool when implemented in chiropractic practice and has found its prognostic value to be limited among patients with acute LBP [49, 51]. To date, to our knowledge, there are no published empirical studies investigating the high risk groups (ID and DYS) classified by the MPI-S instrument or high risk groups classified by the Keele STarT Back Tool [43, 52, 53] or the Örebro Musculoskeletal Screening Tool (ÖMST) [27, 30, 54].

Although valid and reliable, the Swedish version of the MPI instrument (MPI-S) was not designed to be used as a screening instrument in daily clinical practice but rather as a comprehensive research tool. To utilize the aforementioned research findings [20, 40, 41] in clinical practice, a pragmatic and convenient instrument based on the MPI-S with fewer items needs to be developed.

The objective of this study was threefold: (1) To develop a new brief instrument for identifying dysfunctional patients in a clinical setting, with adequate sensitivity, specificity, and receiver operating characteristics. (2) To assess the instrument’s ability to reproduce the previously published effect estimates of MC, and (3) To test the sensitivity, specificity, and receiver operating characteristics in 3 other existing datasets to assess external validity and model transportability across populations.

## Method

To address the first two objectives, data from a recently conducted RCT (developmental dataset 1) was utilized, and to answer the last objective, data from 3 other clinical trials described below (external validation datasets 2, 3, and 4) were used. Karolinska Institutet is the custodian of all the datasets used in this study which have been used and are reported on in previously published papers [19, 20, 40, 41, 55–61]. Only individuals with a complete MPI-S dataset were used in the analysis, thus there were no missing data of the variables used to develop and validate the instrument.

### Design

Objective 1 (instrument development) was addressed using a cross-sectional analysis of the MPI-S data collected at baseline in developmental dataset 1.

Objective 2 (reproduction of effect) was answered using a longitudinal analysis of data from patients who completed the 12-month RCT comparing the intervention (MC) to the control group (developmental dataset 1) with regards to the total number of days with activity limiting LBP for patients classified as dysfunctional at each level of the MAINTAIN instrument. These effect estimates along with the test statistics estimated in objective 1 were used to recommend clinical thresholds for a positive test.

Objective 3 (external validation of the instrument) was answered using a cross-sectional analysis of the MPI-S data collected at baseline in external validation datasets 2, 3, and 4, with the same procedure used in objective 1, to assess external validity and model transportability across populations.

### Populations

Dataset 1 (developmental) was drawn from a randomized controlled trial with a 12-month follow-up period [19, 20, 40, 41] investigating the effectiveness and cost-effectiveness of MC in a population of patients with recurrent or persistent LBP from chiropractic primary care clinics in Sweden. The trial started in 2012 and was concluded in 2016 with a total of 249 patients completing the 12-month study period with a complete MPI-S dataset.

Dataset 2 (validation) was drawn from a large intervention study entitled “Work and Health in the Processing and Engineering Industries” conducted between 2000 and 2003 at four companies in Sweden [55, 56]. Individuals classified as having a high risk of developing chronic disabling neck pain (NP) and/or LBP and long-term sick leave with a comprehensive risk assessment tool were selected for this study.

The third and fourth datasets (validation) came from a large intervention study entitled “Health-economic Evaluation and Rehabilitation (HUR)” conducted between 1994 and 1997 to evaluate multidisciplinary rehabilitation interventions with regards to their effect on sick leave, health-related quality of life, and cost-effectiveness. Dataset 3 was drawn from an observational outcome study nested within the larger HUR project consisting of subjects suffering from neck and low back pain with intermittent sickness absence [57, 59, 61]. Dataset 4 was extracted from a randomized controlled trial also nested within the larger HUR project consisting of subjects with ongoing sickness absence as a result of NP and LBP [58, 60]. Data were collected as part of the baseline assessment at the initial visit to the clinics. The research projects have been described in detail elsewhere [57–60].

The second, third, and fourth datasets were chosen as they represent a broad selection of different populations with LBP with different severity and consequences with regard to the patient’s condition, i.e., working, on intermittent sick leave, or ongoing sick leave. These 4 populations have been compared with regards to psychological characteristics in a previous publication [62].

### Measures

We define discriminative ability as: "How well the test discriminates between two conditions of interest (health and disease; two stages of a disease etc.)". This can be quantified by the measures of diagnostic accuracy such as Sensitivity, Specificity, Positive Predictive Value (PPV), Negative Predictive Value (NPV), Receiver Operating Characteristics (ROC), and Youden’s index [63].

Based on exploratory factor analysis, Turk and Melzac [64] identified 8 items from the original MPI-S instrument that they suggested can be used to develop a brief version of MPI for the purpose of identifying dysfunctional patients. Based on these 8 items, different scoring algorithms were explored to find a working model. During the process, 2 additional items from the “support” dimension were added to improve discrimination between dysfunctional and interpersonally distressed individuals. A final scoring algorithm was decided by considering an optimal compromise between diagnostic accuracy and ease of use in a clinical setting.

### Outcomes

Outcomes of the study were measures of diagnostic accuracy (Sensitivity, Specificity, Positive Predictive Value (PPV), Negative Predictive Value (NPV), Receiver Operating Characteristics (ROC), and Youden’s index) of the MAINTAIN instrument’s ability to classify dysfunctional subjects according to the original classification algorithm used by the original MPI-S instrument (gold standard).

### Analysis

#### Instrument development

Based on 10 items from the original MPI-S questionnaire, 5 dimensions of the patients’ pain experience were recorded: pain severity (Q1 + Q7), *interference* (Q4 + Q8), *life control* (Q15 + Q18), *affective distress* (Q22 + Q20) and *support* (Q5 + Q14). Tentatively, a dysfunctional patient would score high on pain severity, interference, affective distress, support, and low on life control. A summary score was created based on this assumption, adding together the items from the high dimensions, and subtracting the score from the expected low dimension. The following scoring algorithm was created to generate a summative MAINTAIN Score: Q1 + Q7 + Q4 + Q8 − Q15 − Q18 + Q22 + Q20 + Q5 + Q14, ranging from − 12 to 48. Based on this scoring algorithm, each level of the MAINTAIN Score in developmental dataset 1 was analysed as a possible threshold for a dysfunctional classification and compared to the original MPI-S subgroup classification. A positive test would signify a dysfunctional profile and a negative test would signify either an interpersonally distressed or an adaptive coper profile. For each level of the MAINTAIN Score, the proportion of individuals for each subgroup who had a positive test was calculated. This data was graphically depicted to illustrate the relative frequency (%) of true and false positive tests within each subgroup. In addition, a ROC analysis was performed by plotting the binary outcome (AC/ID vs DYS) of the original instrument against a varying discrimination threshold of the MAINTAIN score. Based on the ROC analysis, an estimate the AUC was used to further report the diagnostic accuracy of the instrument. Diagnostic accuracy based on the AUC metrics were defined as: 0.9–1.0 excellent, 0.8–0.9 very good, 0.7–0.8 good, 0.6–0.7 sufficient, 0.5–0.6 bad, < 0.5 test not useful [63].

By analysing test statistics for each level of the instrument, a trichotomization by three identified thresholds based on iterative discussions within the research group were established. First, a lower threshold was identified where most dysfunctional individuals (> 95%) can be identified with a reasonable number of false positive tests with particular attention to AC individuals (< 20%). Second, an upper threshold where most of the AC individuals (> 95%) have been excluded at the expense of a reasonable number of false negative tests with respect to the DYS individuals (< 20%) was identified. Third, a threshold with the optimum cut-off point where equal weight is given to false positive and false negative values was identified. To illustrate how the optimum cut-off point is defined, the diagnostic accuracy of the instrument across different possible discrimination thresholds (each MS score) is reported in Table 2. The final MAINTAIN instrument is reported in Additional file 1.

#### Reproduction of effect estimates

The difference in the total number of days with pain (the primary outcome in the original RCT) between the control and intervention groups in development dataset 1 was estimated to understand how well the instrument could reproduce the effect size in the subgroup previously shown to respond well to MC. Using a general linear regression model, effect estimates were produced for each level of the MAINTAIN instrument (as a threshold for dysfunctional classification). By using the data on clinical outcomes for each level of the instrument along with the data on instrument performance from the development stage, two specific thresholds were identified to classify patients into: low, moderate, and high probability of MC being effective.

#### External validation across different medical settings

Finally, the MAINTAIN instrument was externally validated in 3 separate data sets consisting of patient populations who were experiencing pain resulting in different degrees of activity limitation collected in different medical settings during different time periods. An analysis of relatedness between the developmental dataset and the validation datasets was not performed as the datasets were substantially different in terms of medical settings, demographics, and outcome occurrence. To establish model transportability test statistics (Sensitivity, Specificity, PPV, NPV, ROC and Youden’s index) were estimated and compared across populations.

### Ethical considerations

The project was approved by the Swedish Ethical Review Authority (Ref.: 2019-04505). No new data was collected. Data had been handled and stored in accordance with the tenets of the World Medical Association’s Declaration of Helsinki, and all the subjects had previously signed informed consent forms and agreed to participate in each of the four studies.

## Results

The mean age was similar across all datasets except for the distribution of females which was higher in dataset 1 and lower in dataset 2. Compared to the dataset 1 mean MAINTAIN scores in datasets 3 and 4 were higher, and lower in dataset 2. This is in in line with the rationale described in the method, as the datasets were chosen to represent distinctively different populations with regards to medical setting, status of sick listing and distribution of MPI subgroups. Demographics of datasets 1–4 used in this study are reported in Table 1.

**Table 1:** Patient demographics of the 4 datasets

Variable	Dataset 1 (Development)	Dataset 2 (External validation)	Dataset 3 (External validation)	Dataset 4 (External validation)
Description of recruitment sites and sample characteristics	Primary care “Chiropractic”, not on sick leave	Primary care, not on sick leave with a high risk of chronicity	Secondary care, recurrent sick leave	Secondary care, ongoing sick leave
n	249	128	251	184
Female, n (%)	154 (61.8)	14 (10.9)	140 (55.8)	101 (54.9)
Age, Mean (SD)	43.4 (11.9)	41.5 (9.6)	42.3 (9.3)	43.6 (10.3)
Pain Severity^a^, Mean (SD)	3.3 (1.1)	2.8 (1.3)	3.5 (1.1)	3.7 (1.1)
Interference^a^, Mean (SD)	2.8 (1.3)	2.5 (1.3)	3.8 (1.1)	3.8 (1.0)
Life Control^a^, Mean (SD)	3.5 (1.1)	3.8 (1.1)	3.2 (1.1)	3.1 (1.2)
Affective Distress^a^, Mean (SD)	2.7 (1.3)	2.3 (1.4)	2.6 (1.6)	2.8 (1.5)
Support^a^, Mean (SD)	4.1 (1.6)	4.2 (1.6)	4.6 (1.5)	4.8 (1.6)
Punishing Responses^a^, Mean (SD)	1.1 (1.3)	1.3 (1.3)	1.1 (1.2)	1.0 (1.2)
Solicitous Responses^a^, Mean (SD)	2.7 (1.4)	2.9 (1.4)	3.2 (1.4)	3.1 (1.4)
Distracting responses^a^, Mean (SD)	2.8 (1.4)	3.0 (1.6)	3.1 (1.6)	3.2 (1.6)
MPI-Subgroup, % (AC/ID/DYS)	36.5/24.9/38.6	48.4/25.8/25.8	24.9/24.5/50.6	21.2/25.5/53.3
MAINTAIN Score, − 12 to 48, Mean (SD)	20.0 (8.4)	16.5 (9.7)	23.0 (8.9)	23.5 (8.1)

AC, Adaptive Coper; ID, Interpersonally Distressed; DYS, Dysfunctional; SD, Standard Deviation

^a^Dimensions from the MPI instrument

### Objective 1, instrument development

**Fig. 1:**
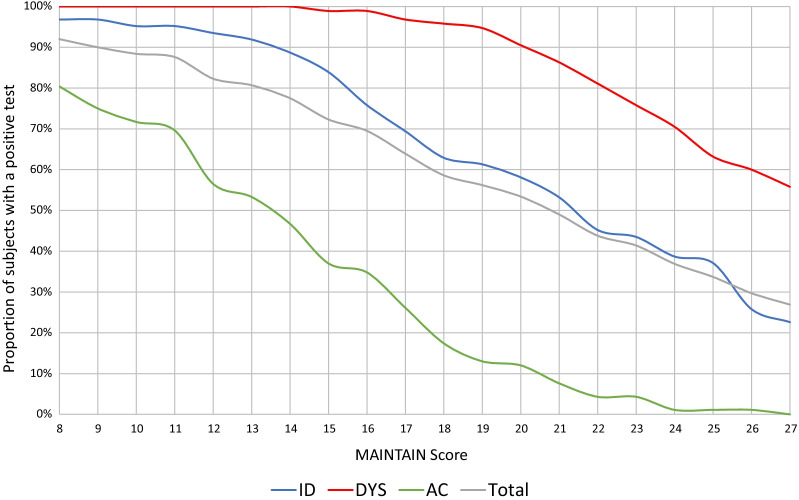
Proportion of individuals classified with the original MPI-S instrument with a positive test in development dataset 1 per level of the MAINTAIN instrument (MAINTAIN Score)

Based on development dataset 1, three different MAINTAIN thresholds were identified. First, a threshold of 18 resulted in a reasonable trade-off where 95.8% of all DYS individuals were correctly classified and only 17.4% of AC individuals were falsely classified as DYS. At this level, 62.9% of ID individuals were falsely classified as DYS. At a score of 18 or higher, 146 out of the 249 patients were classified as dysfunctional with 95.8% sensitivity and 64.3% specificity. Second, at a threshold of 22, 81.1% of all DYS individuals were correctly classified and only 4.3% of AC individuals were falsely classified as DYS. At this level, 45.2% of all ID individuals were falsely classified as DYS. At a score of 22 or higher, 109 were classified as dysfunctional with 81.1% sensitivity and 79.2% specificity. Third, a threshold with the optimum cut-off point where equal weight is given to false positive and false negative values (highest value on the Youden´s index, 0.70) was identified at a MAINTAIN score of 20. At this level, 133 individuals were classified as dysfunctional with 90.5% sensitivity and 69.5% specificity. The AUC was estimated to 0.87 (95% CI 0.83, 0.92; *p* < 0.001). The test statistics for each of these scores of the MAINTAIN instrument in development dataset 1 are reported in Table 2. In Fig. 1, the proportion of individuals classified with the original MPI-S instrument with a positive test per level of the MAINTAIN instrument is reported. The MAINTAIN instrument is presented in Additional file 1.

**Table 2:** Diagnostic accuracy in development dataset 1 using each level of the MAINTAIN instrument as possible discrimination thresholds to classify dysfunctional patients (96 individuals were classified as dysfunctional by the original instrument in this sample)

Development dataset 1 (n = 249)						
MS	n (DYS)	Sensitivity (%)	Specificity (%)	PPV (%)	NPV (%)	Youden’s index
**8**	229	100.0	13.0	41.5	100.0	0.110
**9**	224	100.0	16.2	42.4	100.0	0.130
**10**	220	100.0	18.8	43.2	100.0	0.162
**11**	218	100.0	20.1	43.6	100.0	0.188
**12**	205	100.0	28.6	46.3	100.0	0.201
**13**	201	100.0	31.2	47.3	100.0	0.286
**14**	193	100.0	36.4	49.2	100.0	0.312
**15**	180	98.9	44.2	52.2	98.6	0.364
**16**	173	98.9	48.7	54.3	98.7	0.431
**17**	159	96.8	56.5	57.9	96.7	0.476
*18*	146	95.8	64.3	62.3	96.1	0.533
*19*	140	94.7	67.5	64.3	95.4	0.601
*20*	133	90.5	69.5	64.7	92.2	**0.623**
*21*	122	86.3	74.0	67.2	89.8	0.600
***22***	109	81.1	79.2	70.6	87.1	0.603
***23***	103	75.8	79.9	69.9	84.2	0.603
***24***	92	70.5	83.8	72.8	82.2	0.557
***25***	84	63.2	84.4	71.4	78.8	0.543
***26***	74	60.0	89.0	77.0	78.3	0.476
***27***	67	55.8	90.9	79.1	76.9	0.490

MS, MAINTAIN Score (emphasis represent recommended thresholds); n(DYS), number of individuals classified as dysfunctional by the MAINTAIN instrument at that threshold; PPV, Positive Predictive Value; NPV, Negative Predictive Value

### Objective 2, reproduction of effect

Data on clinical outcomes from development dataset 1, number of days with activity limiting pain (primary outcome), was used to estimate the effect of the intervention (difference between MC and control) for patients classified as Dysfunctional at each score of the MAINTAIN instrument. A positive trend was observed, with a higher effect of MC with a higher MAINTAIN score in a dose–response-like relationship. Patients in the MC-group classified as dysfunctional at a score of 18 or higher, experienced on average 24.4 (95% CI − 0.3, 49.1) fewer days with activity limiting pain compared to dysfunctional patients in the control group, whereas those classified at a score of 22 or higher reported on average 34.5 (95% CI 5.2, 63.7) fewer days with activity limiting pain. Effect estimates for each score of the MAINTAIN instrument are reported in Table 3.

**Table 3:** Effect of maintenance care with a dysfunctional classification for each of the scores 8–27 as possible discrimination thresholds on the MAINTAIN instrument (development dataset 1)

MS	n (DYS)	Difference in total number of days with activity limiting LBP, MC-Control (95% CI), sample 1 (n = 249)
**8**	229	− 13.6 (− 32.1, 5.0)
**9**	224	− 13.3 (− 32.2, 5.7)
**10**	220	− 16.0 (− 34.9, 3.0)
**11**	218	− 16.6 (− 35.8, 2.5)
**12**	205	− 17.7 (− 37.7, 2.4)
**13**	201	− 16.3 (− 36.6, 4.0)
**14**	193	− 19.7 (− 40.5, 1.1)
**15**	180	− 19.6 (− 41.3, 2.1)
**16**	173	− 22.2 (− 44.5, 0.0)
**17**	159	− 22.9 (− 46.3, 0.5)
*18*	146	− 24.4 (− 49.1, 0.3)
*19*	140	− 28.9 (− 53.6, − 4.2)
*20*	133	− 31.5 (− 57.2, − 5.8)
*21*	122	− 32.6 (− 59.8, − 5.4)
***22***	109	− 34.5 (− 63.7, − 5.2)
***23***	103	− 34.1 (− 64.6, − 3.6)
***24***	92	− 29.9 (− 61.6, 1.7)
***25***	84	− 27.2 (− 60.0, 5.6)
***26***	74	− 30.0 (− 66.2, 6.2)
***27***	67	− 25.1 (− 64.0, 13.9)

MS, MAINTAIN Score (emphasis represent recommended thresholds); n(DYS), number of individuals classified as dysfunctional by the MAINTAIN instrument at that threshold

### Objective 3, external validation across different medical settings

In validation dataset 2, at a score of 18 or higher, 61 out of the 128 patients were classified as Dysfunctional with 93.9% sensitivity and 68.4% specificity. At a score of 22 or higher, 41 were classified as dysfunctional with 84.8% sensitivity and 86.3% specificity. In validation dataset 3, at a score of 18 or higher, 183 out of the 251 patients were classified as Dysfunctional with 96.1% sensitivity and 50.8% specificity. At a score of 22 or higher, 148 were classified as Dysfunctional with 85.8% sensitivity and 68.5% specificity. In validation dataset 4, at a score of 18 or higher, 142 out of the 184 patients were classified as dysfunctional with 95.9% sensitivity and 44.2% specificity. At a score of 22 or higher 108 were classified as Dysfunctional with 80.6% sensitivity and 66.3% specificity.

The test showed very good to excellent diagnostic accuracy (ROC characteristics) in all datasets with high AUC estimates that were statistically significant. When considering the optimum cut-off point where equal weight is given to false positive and false negative values (highest value on the Youden´s index) the thresholds differ somewhat in development dataset 1 compared to validation datasets 2 and 3. In validation dataset 4, the highest index (0.451) can be found at a MAINTAIN score of 20 resembling the data from development dataset 1. Whereas the highest index in validation datasets 2 and 3 can be found at a MAINTAIN score of 25 (0.712) and 27 (0.590), respectively.

The test statistics for each score of the MAINTAIN instrument in validation datasets 2 to 4 are reported in Additional files 2, 3 and 4. AUCs for datasets 1 to 4 are reported in Table 4.

**Table 4:** Area under the ROC curve statistics for MAINTAIN instrument in datasets 1–4

Sample	n	AUC (95% CI)
1	249	0.875 (0.833, 0.917)
2	128	0.902 (0.846, 0.958)
3	251	0.844 (0.795, 0.892)
4	184	0.791 (0.724, 0.857)

AUC, area under the ROC curve

## Discussion

To our knowledge, this is the first study to develop and psychometrically assess a brief version of MPI-S with the purpose of identifying dysfunctional subjects suitable for MC in a clinical setting, see Additional file 1. The MAINTAIN instrument has the potential of being an effective clinical decision aid allowing clinicians to acquire a relevant score of psychological distress quickly with a low administrative load. The instrument has displayed an acceptable trade-off between sensitivity and specificity across contexts and populations (model transportability) and can be used with different thresholds depending on the rate of false positive tests that can be accepted in the clinical encounter. The instrument has exhibited high ROC statistics with scores ranging between 0.79 and 0.90, thus, suggesting very good to excellent diagnostic accuracy [63, 65]. Rather than using a single threshold with a dichotomous outcome we recommend 3 ranges to illustrate that the instrument represents a scale where the likelihood of a good outcome from MC changes in a dose–response-like relationship as reported in Table 3. We suggest clinicians use the instrument as one parameter in a broader assessment strategy where previous pain history, initial treatment effectiveness, ability to perform active strategies, and patient preferences should be considered.

In the present work, datasets were collected from different patient populations in different medical settings which has allowed for a robust evaluation and introspection of the instrument’s diagnostic accuracy. Also, by verifying the stratification procedure using clinical outcomes, the clinical usefulness of the instrument is assessed. We considered the data from this trial to be robust and trustworthy, and the MAINTAIN instrument adds a valuable contribution by informing clinical decision-making by deepening the understanding of the psychological characteristics of the patient’s condition. The advantage of a brief instrument with a simple scoring algorithm is the possibility to employ it during the clinical encounter and being able to score while the patient is present. This allows the clinician to promptly get an overview of the psychological dimensions of the patient’s pain experience as well as an opportunity to dwell into each of the 5 dimensions, *pain severity, interference, life control, affective distress,* and *support,* with probing follow-up questions. In addition to deepening the understanding of the patient’s pain condition, this information can be used to establish treatment goals or adapt the intervention to better align with the patients’ needs and preferences.

Patients included in sample 1, where the effect and cost-effectiveness of MC were investigated, were included only if they reported recurrent or persistent pain, and the long-term effect over 12 months was studied. This may explain the very good to excellent discriminant ability of the MAINTAIN instrument in contrast to the research done on the Keele STarT Back Tool. Thus, psychometric properties, such as validity, may not pertain to an instrument as such; rather, they are a feature of the construal of the results generated from a contextual study [66].

In objective 2, the difference in the total number of days with pain was used to reproduce the effect sizes of the intervention in the new subgroups defined by the thresholds of the MAINTAIN instrument. This outcome has been used in several clinical trials and the measure has been shown to correlate well with pain intensity [67]. However, there is currently no study that has investigated minimally clinically relevant changes in the outcome, and it is therefore difficult to draw firm conclusions on the clinical relevance.

The cross-sectional design used to develop this instrument, naturally, does not allow us to explore the measurement over time and represents a weakness. Additional cross-sectional and longitudinal data on reliability estimates that evaluate the stability, internal consistency, interrater reliability, and responsiveness of the MAINTAIN instrument would further inform on applicability. Also, the datasets used to validate the instrument are quite old, therefore it is possible perceptions and attitudes towards pain in general may have changed over time in the societal context. Whether this has had any impact on how patients have scored the original MPI-s instrument is unclear. Future research should investigate the use of the MAINTAIN instrument in a clinical setting in an implementation trial by comparing a stratified MC approach using the MAINTAIN instrument and comparing it with standard care, exercise interventions, and digital consultations. Also, it would be valuable to contrast and compare the thresholds to the Keele STarT Back Tool and the Örebro Musculoskeletal Screening Pain Questionnaire (ÖMSPQ) to further explore the construct validity and usefulness of the MAINTAIN tool to populations with recurrent or persistent LBP. Such data could shed light on the possibility of using these instruments as alternative decision-making aids for identifying patients suitable for MC.

## Conclusion

The MAINTAIN instrument is a brief cinical tool with a simple scoring algorithm that has a very good to excellent diagnostic accuracy with regard to selecting dysfunctional patients in a clinical setting. By using 2 thresholds, patients can be categorized into “low probability”, “moderate probability” and “high probability” of having a good outcome from maintenance care for LBP. The diagnostic accuracy is similar and high across populations with minor differences in optimal thresholds for identifying dysfunctional individuals. Implementing the MAINTAIN instrument has the potential of improving outcomes by identifying dysfunctional high-risk patients early in the clinical course and stratifying them to appropriate interventions with a higher chance of treatment success.

## Supplementary Information

**Additional file 1. Figure S1**: The MAINTAIN instrument.

**Additional file 2. Table S1**: Diagnostic accuracy in dataset 2 using each level of the MAINTAIN instrument as possible discrimination thresholds to classify dysfunctional patients.

**Additional file 3. Table S2**: Diagnostic accuracy in dataset 3 using each level of the MAINTAIN instrument as possible discrimination thresholds to classify dysfunctional patients.

**Additional file 4. Table S3**: Diagnostic accuracy in dataset 4 using each level of the MAINTAIN instrument as possible discrimination thresholds to classify dysfunctional patients.

## Data Availability

Data cannot be shared publicly for legal reasons. Data will be available upon request after permission is granted by the Swedish Ethical Review Authority: kansli@stockholm.epn.se. Inquiries for data access should first be sent to irene.jensen@ki.se, who will then contact the ethics board for permission to openly share the data.

## Abbreviations

AC: Adaptive coper
DYS: Dysfunctional
ID: Interpersonally distressed
LBP: Low back pain
MC: Chiropractic maintenance care
MPI-S: Swedish version of West Haven-Yale Multidimensional Pain Inventory
