# Supplementary material for: Development and evaluation of the MAINTAIN instrument, selecting patients suitable for secondary or tertiary preventive manual care: the Nordic maintenance care program
Source: Chiropr Man Therap. 2022 Mar 17;30:15. doi: 10.1186/s12998-022-00424-6 (PMC8932000; doi:10.1186/s12998-022-00424-6)
Supplement: Supplementary file 1 — Additional file 1. Figure S1: The MAINTAIN instrument. [file 12998_2022_424_MOESM1_ESM.pdf]

In the following 10 questions, you will be asked to describe your pain and how it affects your life. Under each question is a scale to record your answer. Read each question carefully and then check a number on the scale under that question to indicate how that specific question applies to you.

MAINTAIN
Maintenance Care Instrument

Question									
**1. Rate the level of your pain at the present moment.**									+ PS
No pain	☐ 0	☐ 1	☐ 2	☐ 3	☐ 4	☐ 5	☐ 6	Very intense pain	
**2. How much has your pain changed the amount of satisfaction or enjoyment you get from participating in social and recreational activities?**									+ I
No change	☐ 0	☐ 1	☐ 2	☐ 3	☐ 4	☐ 5	☐ 6	Extreme change	
**3. During the past week, how tense or anxious have you been?**									+ AD
Not at all tense or anxious	☐ 0	☐ 1	☐ 2	☐ 3	☐ 4	☐ 5	☐ 6	Extremely tense or anxious	
**4. How much has your pain changed your ability to participate in recreational and other social activities?**									+ I
No change	☐ 0	☐ 1	☐ 2	☐ 3	☐ 4	☐ 5	☐ 6	Extreme change	
**5. During the past week, how much do you feel that you've been able to deal with your problems?**									- LC
Not at all	☐ 0	☐ 1	☐ 2	☐ 3	☐ 4	☐ 5	☐ 6	Extremely well	
**6. On the average, how severe has your pain been during the last week?**									+ PS
Not at all severe	☐ 0	☐ 1	☐ 2	☐ 3	☐ 4	☐ 5	☐ 6	Extremely severe	
**7. How supportive or helpful is your spouse (significant other) to you in relation to your pain?**									+ S
Not at all supportive	☐ 0	☐ 1	☐ 2	☐ 3	☐ 4	☐ 5	☐ 6	Extremely supportive	
**8. During the past week, how successful were you in coping with stressful situations in your life?**									- LC
Not at all successful	☐ 0	☐ 1	☐ 2	☐ 3	☐ 4	☐ 5	☐ 6	Extremely successful	
**9. During the past week, how irritable have you been?**									+ AD
Not at all irritable	☐ 0	☐ 1	☐ 2	☐ 3	☐ 4	☐ 5	☐ 6	Extremely irritable	
**10. How attentive is your spouse (significant other) to your pain problem?**									+ S
Not at all attentive	☐ 0	☐ 1	☐ 2	☐ 3	☐ 4	☐ 5	☐ 6	Extremely attentive	
-12 – 17 = Red, 18 – 21 = Yellow, 22 - 48 = Green								***Total:***	

Procedure

Consider the following questions when planning care for the long-term management of recurrent or persistent spinal pain.

Question	Yes	No
1. Has the patient experienced the pain before (is it recurrent)?		
2. Has the patient experienced more than 30 days of pain the previous 12 months?		
3. Does the patient have a Maintain Score of 18 or more (yellow or green candidate) at the initial consultation?		
4. Do the patient respond well to manual care (a clear improvement within the first 4 visits)?		

- If the answers to the following 4 questions are YES consider maintenance care as part of a long-term management of the patients spinal pain.
- Initially explore the feasibility of physical activity/exercise as the main intervention for the long-term management.
- If a physical activity/exercise is not effective, not feasible or not preferred by the patient discuss maintenance care as a secondary (in addition to) or primary (instead of) management strategy depending on the patient's clinical status, life circumstances and preferences.
- If the patient scores 18-21, MC may be cost effective and should only be recommended if there is strong preference on behalf of the patient.
- If the patient scores 22-48, MC is likely effective and probably cost-effective and can be recommended.
- Focus on reassuring and empowering messages with the aim of improving self-management strategies and life control while reducing kinesofobia, anxiety and catastrophic thought patterns.

Developing a treatment plan

In addition to generating a maintain score the scores on the individual questions can be used to identify specifically challenging areas to build the treatment plan around. Identify in which of the 5 domains has the highest scores i.e. where the condition has the largest negative impact on the patient's quality of life. Use further probing questions to deepen the understanding by identify how, why, and when they experience this as a problem.

- **Pain Severity (questions 1 and 6)**
  - *When do they experience the pain/what does the trajectory look like?*
  - *When is it worst/best?*
  - *What alleviates/provokes?*
- **Interference (questions 2 and 4)**; how and when the pain affects the:
  - *Ability to work?*
  - *Ability to perform hobbies, activities, and level of activity?*
  - *Ability to household work, social activities, relationships (family, friends, co-workers)?*
  - *Ability to plan activities?*
  - *Ability to experience satisfaction/joy?*
  - *Fear of movement (believes the problem will get worse with load/activities/work)?*
- **Life Control (questions 5 and 8);** how and when the pain affects the ability to:
  - *Control the daily life?*
  - *Handle everyday problems and stressful situations?*
  - *Control/manage pain with exercises, movement, behaviors, or medication?*
- **Affective Distress (questions 3 and 9),** how and when the patients/patient:
  - *Mood/emotional state is affected?*
  - *Gets irritated and tensed?*
  - *Gets worried or scared?*
- **Support (questions 7 and 10),** when and how significant others:
  - *Support the patient when there is a specific need?*
  - *Considers the needs of the patient?*

Together with the patient discuss how to solve these issues. Use the solutions as an integral part of the treatment plan. Use these items along with other outcomes as a means to explore effectiveness of the care plan.
