# Supplementary material for: Development and evaluation of the MAINTAIN instrument, selecting patients suitable for secondary or tertiary preventive manual care: the Nordic maintenance care program
Source: Chiropr Man Therap. 2022 Mar 17;30:15. doi: 10.1186/s12998-022-00424-6 (PMC8932000; doi:10.1186/s12998-022-00424-6)
Supplement: Supplementary file 2 — Additional file 2. Table S1: Diagnostic accuracy in dataset 2 using each level of the MAINTAIN instrument as possible discrimination thresholds to classify dysfunctional patients. [file 12998_2022_424_MOESM2_ESM.docx]

**Supplementary file 2:** Diagnostic accuracy in dataset 2 using each level of the MAINTAIN instrument as possible discrimination thresholds to classify dysfunctional patients

| **Dataset 2 (n=128)** | | | | | |
| --- | --- | --- | --- | --- | --- |
| **MS** | **Sensitivity (%)** | **Specificity (%)** | **PPV (%)** | **NPV (%)** | **Youden’s index** |
| 8 | 100.0 | 22.1 | 30.8 | 100.0 | 0.158 |
| 9 | 100.0 | 27.4 | 32.4 | 100.0 | 0.179 |
| 10 | 100.0 | 31.6 | 33.7 | 100.0 | 0.200 |
| 11 | 100.0 | 37.9 | 35.9 | 100.0 | 0.221 |
| 12 | 100.0 | 41.1 | 37.1 | 100.0 | 0.274 |
| 13 | 97.0 | 48.4 | 39.5 | 97.9 | 0.316 |
| 14 | 93.9 | 53.7 | 41.3 | 96.2 | 0.379 |
| 15 | 93.9 | 56.8 | 43.1 | 96.4 | 0.411 |
| 16 | 93.9 | 61.1 | 45.6 | 96.7 | 0.454 |
| 17 | 93.9 | 67.4 | 50.0 | 97.0 | 0.476 |
| 18 | 93.9 | 68.4 | 50.8 | 97.0 | 0.508 |
| 19 | 90.9 | 71.6 | 52.6 | 95.8 | 0.550 |
| 20 | 90.9 | 78.9 | 60.0 | 96.2 | 0.613 |
| 21 | 84.8 | 82.1 | 62.2 | 94.0 | 0.624 |
| 22 | 84.8 | 86.3 | 68.3 | 94.3 | 0.625 |
| 23 | 78.8 | 88.4 | 70.3 | 92.3 | 0.699 |
| 24 | 69.7 | 89.5 | 69.7 | 89.5 | 0.670 |
| 25 | 60.6 | 90.5 | 69.0 | 86.9 | **0.712** |
| 26 | 54.5 | 93.7 | 75.0 | 85.6 | 0.672 |
| 27 | 48.5 | 94.7 | 76.2 | 84.1 | 0.592 |

MS, MAINTAIN Score (colours represent recommended thresholds); n(DYS), number of individuals classified as dysfunctional by the MAINTAIN instrument at that threshold; PPV, Positive Predictive Value; NPV, Negative Predictive Value
