# Supplementary material for: Development and evaluation of the MAINTAIN instrument, selecting patients suitable for secondary or tertiary preventive manual care: the Nordic maintenance care program
Source: Chiropr Man Therap. 2022 Mar 17;30:15. doi: 10.1186/s12998-022-00424-6 (PMC8932000; doi:10.1186/s12998-022-00424-6)
Supplement: Supplementary file 3 — Additional file 3. Table S2: Diagnostic accuracy in dataset 3 using each level of the MAINTAIN instrument as possible discrimination thresholds to classify dysfunctional patients. [file 12998_2022_424_MOESM3_ESM.docx]

**Supplementary file 3:** Diagnostic accuracy in dataset 3 using each level of the MAINTAIN instrument as possible discrimination thresholds to classify dysfunctional patients

| **Dataset 3 (n=251)** | | | | | |
| --- | --- | --- | --- | --- | --- |
| **MS** | **Sensitivity (%)** | **Specificity (%)** | **PPV (%)** | **NPV (%)** | **Youden’s index** |
| 8 | 100.0 | 5.6 | 52.0 | 100.0 | 0.040 |
| 9 | 100.0 | 6.5 | 52.3 | 100.0 | 0.051 |
| 10 | 100.0 | 10.5 | 53.4 | 100.0 | 0.070 |
| 11 | 100.0 | 13.7 | 54.3 | 100.0 | 0.083 |
| 12 | 100.0 | 18.5 | 55.7 | 100.0 | 0.098 |
| 13 | 100.0 | 21.8 | 56.7 | 100.0 | 0.115 |
| 14 | 100.0 | 27.4 | 58.5 | 100.0 | 0.130 |
| 15 | 99.2 | 34.7 | 60.9 | 97.7 | 0.161 |
| 16 | 98.4 | 40.3 | 62.8 | 96.2 | 0.193 |
| 17 | 96.9 | 47.6 | 65.4 | 93.7 | 0.231 |
| 18 | 96.1 | 50.8 | 66.7 | 92.6 | 0.261 |
| 19 | 95.3 | 56.5 | 69.1 | 92.1 | 0.296 |
| 20 | 92.1 | 62.1 | 71.3 | 88.5 | 0.345 |
| 21 | 89.0 | 64.5 | 72.0 | 85.1 | 0.397 |
| 22 | 85.8 | 68.5 | 73.6 | 82.5 | 0.431 |
| 23 | 82.7 | 71.0 | 74.5 | 80.0 | 0.486 |
| 24 | 76.4 | 75.8 | 76.4 | 75.8 | 0.531 |
| 25 | 69.3 | 78.2 | 76.5 | 71.3 | 0.560 |
| 26 | 63.0 | 84.7 | 80.8 | 69.1 | 0.577 |
| 27 | 55.9 | 86.3 | 80.7 | 65.6 | **0.590** |

MS, MAINTAIN Score (colours represent recommended thresholds); n(DYS), number of individuals classified as dysfunctional by the MAINTAIN instrument at that threshold; PPV, Positive Predictive Value; NPV, Negative Predictive Value
