# Supplementary material for: Development and evaluation of the MAINTAIN instrument, selecting patients suitable for secondary or tertiary preventive manual care: the Nordic maintenance care program
Source: Chiropr Man Therap. 2022 Mar 17;30:15. doi: 10.1186/s12998-022-00424-6 (PMC8932000; doi:10.1186/s12998-022-00424-6)
Supplement: Supplementary file 4 — Additional file 4. Table S3: Diagnostic accuracy in dataset 4 using each level of the MAINTAIN instrument as possible discrimination thresholds to classify dysfunctional patients. [file 12998_2022_424_MOESM4_ESM.docx]

**Supplementary file 4:** Diagnostic accuracy in dataset 4 using each level of the MAINTAIN instrument as possible discrimination thresholds to classify dysfunctional patients

| **Dataset 4 (n=184)** | | | | | |
| --- | --- | --- | --- | --- | --- |
| **MS** | **Sensitivity (%)** | **Specificity (%)** | **PPV (%)** | **NPV (%)** | **Youden’s index** |
| 8 | 100.0 | 5.8 | 54.7 | 100.0 | 0.198 |
| 9 | 100.0 | 7.0 | 55.1 | 100.0 | 0.244 |
| 10 | 100.0 | 11.6 | 56.3 | 100.0 | 0.267 |
| 11 | 100.0 | 14.0 | 57.0 | 100.0 | 0.269 |
| 12 | 100.0 | 16.3 | 57.6 | 100.0 | 0.339 |
| 13 | 100.0 | 19.8 | 58.7 | 100.0 | 0.401 |
| 14 | 100.0 | 24.4 | 60.1 | 100.0 | 0.427 |
| 15 | 100.0 | 26.7 | 60.9 | 100.0 | 0.401 |
| 16 | 99.0 | 27.9 | 61.0 | 96.0 | 0.428 |
| 17 | 99.0 | 34.9 | 63.4 | 96.8 | 0.469 |
| 18 | 95.9 | 44.2 | 66.2 | 90.5 | 0.462 |
| 19 | 93.9 | 48.8 | 67.6 | 87.5 | 0.443 |
| 20 | 87.8 | 52.3 | 67.7 | 78.9 | 0.451 |
| 21 | 84.7 | 58.1 | 69.7 | 76.9 | 0.415 |
| 22 | 80.6 | 66.3 | 73.1 | 75.0 | 0.334 |
| 23 | 77.6 | 68.6 | 73.8 | 72.8 | 0.317 |
| 24 | 74.5 | 69.8 | 73.7 | 70.6 | 0.258 |
| 25 | 68.4 | 76.7 | 77.0 | 68.0 | 0.210 |
| 26 | 61.2 | 80.2 | 77.9 | 64.5 | 0.182 |
| 27 | 52.0 | 81.4 | 76.1 | 59.8 | 0.146 |

MS, MAINTAIN Score (colours represent recommended thresholds); n(DYS), number of individuals classified as dysfunctional by the MAINTAIN instrument at that threshold; PPV, Positive Predictive Value; NPV, Negative Predictive Value
